# Wellbeing at work among GPs working in multidisciplinary primary care teams: a cross-sectional study

**DOI:** 10.3399/BJGPO.2024.0201

**Published:** 2026-01-14

**Authors:** Christine Cohidon, Adeline Cachou de Camaret, Nicolas Senn, Pascal Wild

**Affiliations:** 1 Department of Family Medicine, Center for Primary Care and Public Health,University of Lausanne, Vaud, Switzerland; 2 PW Consulting, Laxou, France

**Keywords:** general practitioners, typology, wellbeing, job satisfaction, primary health care

## Abstract

**Background:**

Transforming primary care (PC) through the development of multidisciplinary teams can represent a challenge in terms of occupational wellbeing.

**Aim:**

To investigate associations between occupational stress, job satisfaction among GPs, and the professional composition of PC teams.

**Design & setting:**

We conducted a secondary analysis of the data from 11 Western countries that participated in the 2019 Commonwealth Fund International Health Policy Survey of Primary Care Physicians (*n* = 13 200).

**Method:**

PC practice types (*n* = 5) were defined in a previous study, based on their composition of healthcare professionals, which were as follows: traditional; multidisciplinary; nurse-centred; psychologist-centred; and physiotherapist-centred models. Using ordered logistic regression analysis, we assessed associations between the five practice models and two GP-reported indicators of wellbeing at work: job satisfaction and occupational stress.

**Results:**

Working in multidisciplinary teams, when compared with traditional (GP-centred) practice, was associated with higher occupational wellbeing, both through lower occupational stress (odds ratio [OR] 0.77, 95% confidence interval [CI] = 0.68 to 0.87) and greater job satisfaction (OR 1.43, 95% CI = 1.26 to 1.62). This positive association was also observed in psychologist-centred practices for occupational stress (OR 0.81, 95% CI = 0.71 to 0.93) and for job satisfaction (OR 1.24, 95% CI = 1.09 to 1.42). Working in nurse-centred practices was associated with greater satisfaction but only in the smallest practices (OR 1.59, 95% CI = 1.14 to 2.22) with <1.4 full-time equivalent (FTE) GPs.

**Conclusion:**

Positive associations between multidisciplinary PC teams and occupational wellbeing are important results for the future of healthcare systems in Western countries, providing interesting avenues for improvements for healthcare professionals and policymakers.

## How this fits in

Healthcare systems have to adapt rapidly to population needs. The major evolution in primary care (PC) involves changes in work organisation, especially the development of multidisciplinary teams. This evolution from a physician-centred culture to a team-based one might also represent a challenge to GPs’ wellbeing. This study showed that working in multidisciplinary teams was associated with higher GPs’ wellbeing, both through lower occupational stress and higher job satisfaction.

## Introduction

In the context of shortages and recruitment crises in many countries, maintaining healthcare professionals’ health and wellbeing has become a growing concern for public health decision-makers.^1,2^ In primary care (PC), GPs have been the subjects of numerous studies describing their exposure to occupational stress and its consequences, mainly in terms of burnout.^3–5^ Exposure to work overload and, in particular, administrative overload are among the factors most often found to generate work-related stress and dissatisfaction.^6–10^

After the Alma-Ata conference and Astana conferences,^11^ Western countries adopted strategies for strengthening PC to meet their populations’ growing needs. The major evolution involves changes in work organisation, especially the development of multidisciplinary teams.^12,13^ The introduction of PC teams has often been presented as an innovation that should improve GPs’ working conditions^2^ through reduced workloads, the delegation of activities such as administrative tasks and shared responsibilities.^14,15^ If we refer to epidemiological models of work stress, such as Karasek’s and Siegrist’s, in which quantitative and qualitative workload (’job demand’ for Karasek’s model^16^ and ‘efforts’ for Siegrist’s model),^17,18^ as well as social support are major components, then it’s clear that the shift from a physician-centred model to a multidisciplinary one can have a positive impact on GP wellbeing. However, implementing major changes also requires considerable work by GPs with the transition from a physician-centred culture to a team-based one (organisational changes to workflows, role redistributions, recruitment, and training of new professional staff).^19–21^ In addition, obstacles to integrating other professionals into PC practices include GPs’ fears of losing exclusivity in the patient–physician therapeutic relationship and their difficulties accepting the transfer of tasks to others, both crucial issues at the core of a GP’s autonomy and, therefore, the very meaning of their profession.^22^

Despite many studies on how new models of care affect patient outcomes, the literature on how it affects GPs’ job satisfaction and occupational stress remains sparse and sometimes contradictory.^21,23–26^ While conceptual models and several studies pointed to an improvement in GPs' wellbeing during teamwork,^26–29^ several recent studies find no evidence of this improvement. In 2018, Peikes *et al* founded no effect of PC transformation on GPs’ satisfaction and burnout.^25^ More specifically, the study by Casalino *et al* ^21^ reported that physicians practising in a teamlet (pair comprising GP-one staff member) were more likely to report burnout compared with physicians practising neither in a teamlet nor in a team. They suggested that this question be explored in greater depth, depending on the type of team. Finally, according to Riisgaard *et al*, from the GPs’ perspectives, the relationship with the patients was influenced negatively as the changes in the working structure challenged the continuity of care. Additionally, they experienced an increase in the workload caused by the delegation of tasks as they had to supervise their staff.^22^

The present study aimed to investigate associations between GPs’ occupational stress and job satisfaction and the professional composition of PC teams in 11 Western countries.

## Method

This study was a secondary analysis of data from the 2019 Commonwealth Fund International Health Policy Survey of Primary Care Physicians. The global aim of the survey was to collect PC providers’opinions on their country’s health system; and more precisely, to observe the preparedness to manage the care of patients with complex needs in the participant countries. The survey also collected information about GPs’ satisfaction with aspects of their practice and occupational stress.^7,30^ The 2019 survey’s study protocol has been detailed elsewhere.^31,32^

### Population and data collection

GPs from Australia, Canada, France, Germany, the Netherlands, New Zealand, Norway, Sweden, Switzerland, the UK, and the US participated in the survey’s 2019 edition.^31,32^ National samples of practising PC physicians (GPs or family physicians, internists and paediatricians also in Germany, Switzerland and the US) were randomly drawn from government or private lists in each country or, for France, from a nationally representative panel of PC physicians.^32^ Physicians were surveyed between January and June 2019 by post, via the internet, or by telephone.^31,32^

### Data

A common questionnaire was reviewed by experts in each country and translated to ensure comparability across countries. As the questionnaire covered many subdomains, it was impossible to integrate a complete validated tool for each of them. Consequently, the questionnaire included a minimum number of questions for each subdomain. This was particularly true of wellbeing outcomes, which were assessed using a question developed for the study. Occupational stress was assessed using the question, ’How stressful, if at all, is your job as a GP?’ This was answered on a five-point Likert scale (from ’Extremely stressful’ to ’Not at all stressful‘). Job satisfaction was explored using the item, ‘How satisfied are you with practising medicine?’, and it was answered on a five-point Likert scale (from‘Extremely satisfied’ to ‘Not at all satisfied’).

**Figure 1.**
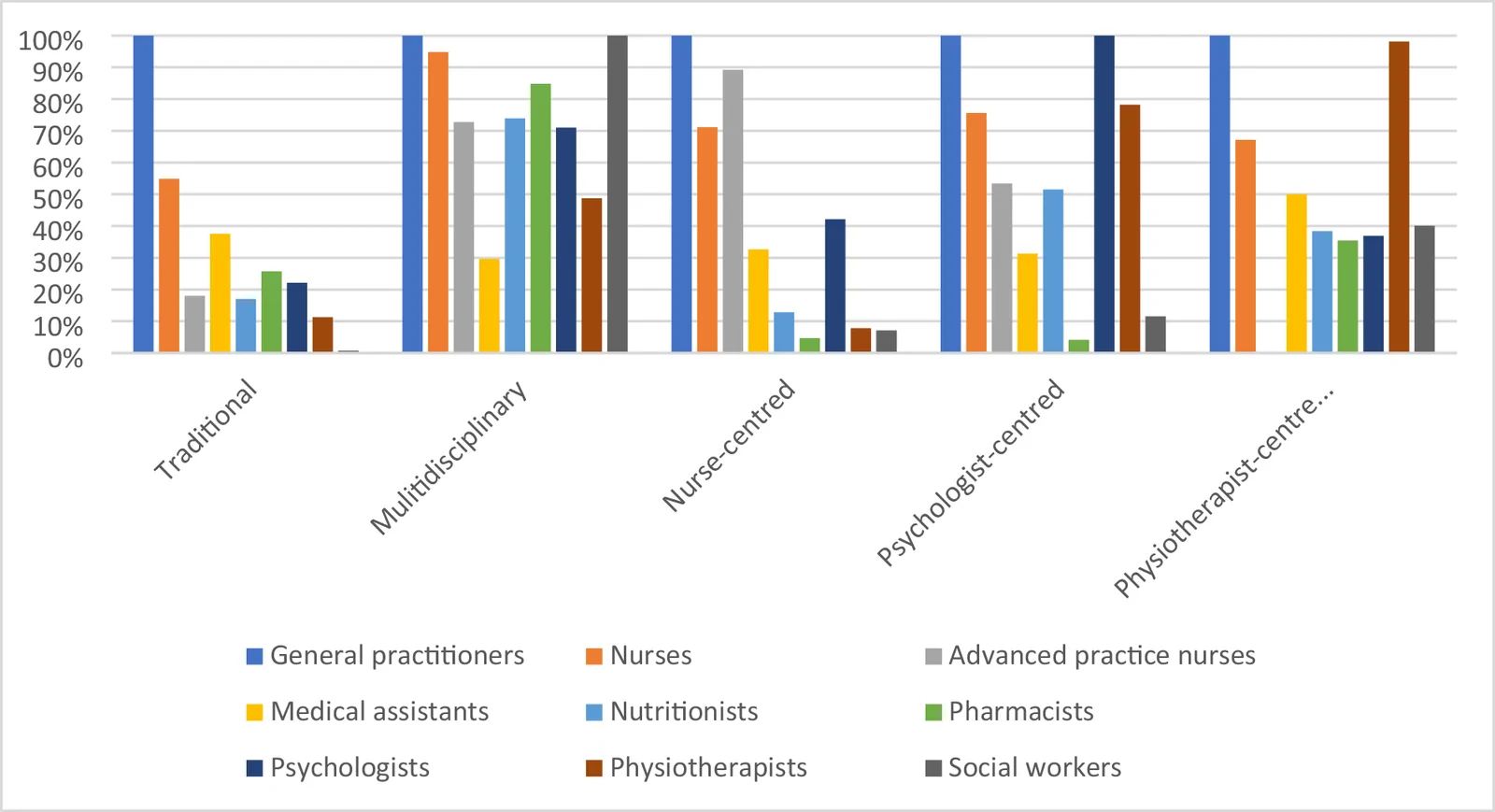
Staff composition of different PC practice types¥^¥^ (in addition to GPs) (*n* = 13 027 GPs). *Percentage of practices working with each professional for each practice type ^¥^The traditional type represents 52% of the practices, the multidisciplinary type = 12%, the nurse-centred type = 12%, the psychologist-centred type = 19%, the physiotherapist type = 5%

Information on the professional composition of PC teams stemmed from another study based on the same dataset^33^. In summary, we created a classification of different PC staff compositions using the question, ’In your main practice, do the following healthcare professionals work on your team to provide care for your patients?’ The response options were ‘Yes’ or ‘No’ for each of the following categories of staff: nurse(s); advanced practice nurse(s) (for example, nurse practitioner[s]); physician(s) or medical assistant(s); nutritionist(s) or dietician(s); pharmacist(s); psychologist(s) or mental health professional(s); physical therapist(s) or physiotherapist(s); and social worker(s). First, we used a hierarchical clustering algorithm, with complete linkage and a simple matching similarity coefficient, to characterise the different types of organisations in each of the 11 countries according to their compositions of professionals (in addition to GPs). Five clusters were identified. Second, the different types of organisations were interpreted and denominated, according to the types of professionals identified, as traditional, multidisciplinary, nurse-centred, psychologist-centred, and physiotherapist-centred models. Figure 1 describes the five clusters according to their compositions of different healthcare professionals (percentage of practices working with each professional in each type of practice). Please note that, according to the design of the study, the GP was systematically part of the team.

Finally, we also selected variables for use as confounding factors in our statistical regression models; that is, physicians’ sociodemographic characteristics (sex, age category), practice location (rural or urban area), consultation durations (in four classes), and GP workforce size (full-time equivalent [FTE], FTE in quartiles, this last variable was lacking for Sweden).

### Analysis

The Commonwealth Fund provided country-specific sampling weights that accounted for the potential over-representation of GPs in relation to certain factors (that is, sex, age, practice location, medical specialty). This allowed a more representative sample to obtain for epidemiological purpose. All the analyses were weighted using these sampling weights.

First, descriptive statistics characterising the prevalence of occupational stress and job satisfaction in each country were produced (weighted data). Second, we modelled the two dependent variables of occupational stress and job satisfaction using ordered logistic regressions as a function of the type of PC practice. Selected physician and practice characteristics were considered, too, including the physician’s sex and age category, practice location, consultation duration, FTE GPs, and country. Each independent variable was introduced separately and, in a second stage, jointly to build final models (one final model for each dependent variable). The five-point Likert scales were transformed into three levels of exposure ’low’, ‘moderate’, and ‘high’. Finally, as we suspected that the type of practice typology might have a differential effect depending on the number of FTE GPs,^34,35^ we conducted stratified analyses for each class of FTE GPs. All statistical analyses were performed using Stata software (version 17.0).

## Results

Table 1 shows the sample characteristics for each participating country. Response rates ranged from 14% in Australia to 49% in the Netherlands, with a total of 13 200 GPs completing the survey questionnaire (Table 1).

**Table 1.** GPs’ personal characteristics, job satisfaction, and occupational stress (weighted data)

	Australia	Canada	France	Germany	Netherlands	New Zealand	Norway	Sweden	Switzerland	**UK**	US	Total
**Final sample size**	500	2569	1287	809	788	503	661	2411	1095	1001	1576	13 200
**Response rate (%)**	14.5	39.3	20.0	14.7	48.7	16.2	33.8	42.2	42.8	26.8	21.2	29.1
**Sex (%) Male**	54.9	54.0	62.5	54.6	47.7	44.9	56.2	48.8	59.4	53.9	55.1	53.9
**Female**	45.1	45.9	37.5	45.3	52.2	55.0	43.7	51.1	40.5	46.0	44.8	46.0
**Age (%)<35–44 (years)**	39.0	34.2	19.2	1.7	40.8	32.3	46.8	39.6	20.3	57.5	30.8	34.0
45–64	46.8	50.3	62.7	66.5	56.9	55.5	44.2	47.4	62.2	36.2	51.3	52.2
>65	14.2	15.3	17.9	16.7	2.1	12.1	8.8	12.9	17.4	6.2	17.7	13.7
**GPs’ job satisfaction (%)**												
Low	4.4	11.9	8.7	12.2	5.5	7.1	7.9	11.8	1.6	15.0	16.3	10.4
Moderate	34.1	43.2	59.1	44.5	40.4	39.6	31.4	35.6	29.0	46.5	41.2	41.1
High	61.6	44.7	32.2	43.3	54.1	53.3	60.7	52.6	69.4	38.5	42.8	48.5
**GPs’ occupational stress (%)**												
Low	15.9	10.3	12.1	11.4	13.3	13.3	7.9	6.4	15.3	5.1	9.9	10.2
Moderate	54.7	43.9	49.6	36.1	55.3	44.9	47.9	28.3	47.2	35.3	37.1	41.2
High	29.4	45.8	38.3	52.5	31.3	41.8	44.2	65.3	37.6	59.6	53.1	48.5

### Occupational stress and job satisfaction among GPs

**Figure 2.**
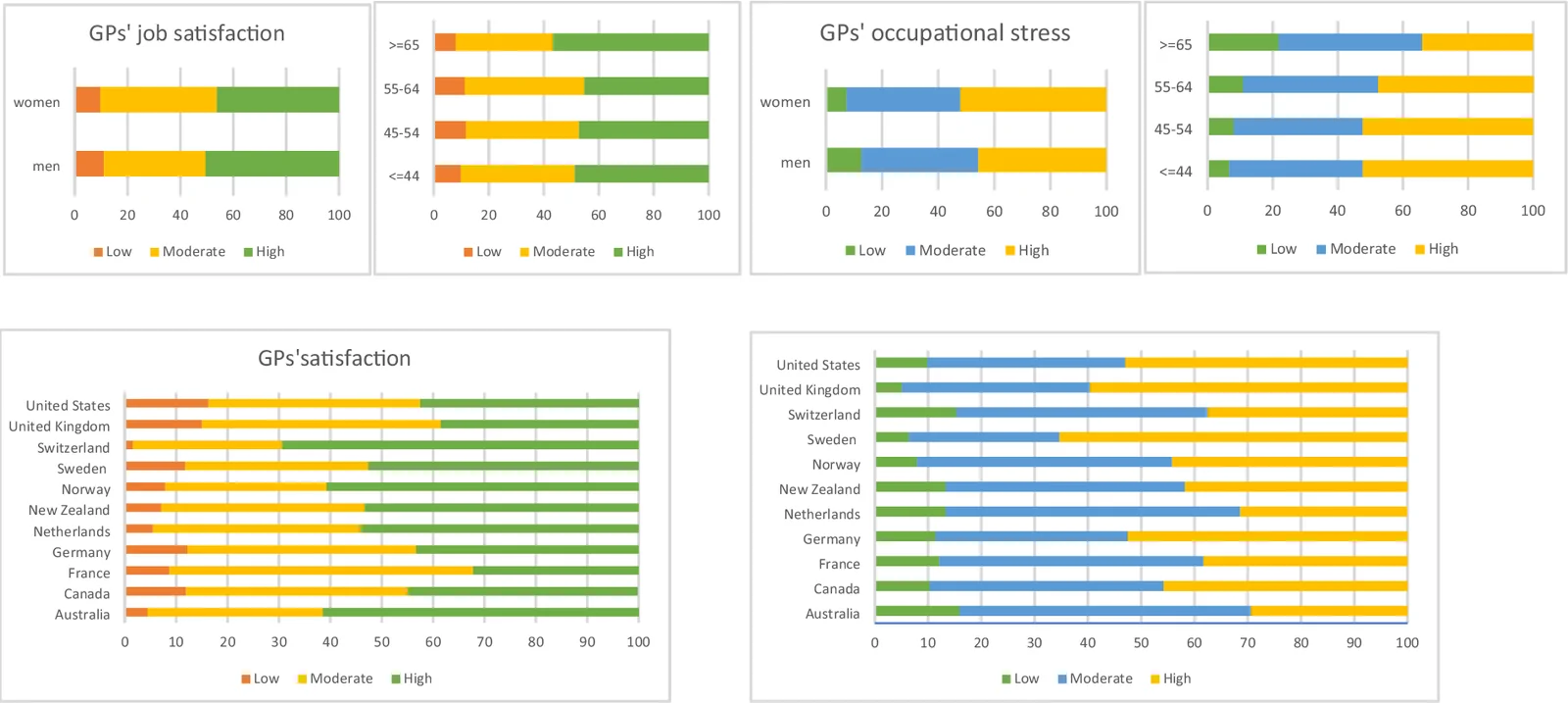
Job satisfaction and occupational stress (%) as reported by GPs, according to their sex and age in 11 Western countries

About half of the GPs (48%) declared being exposed to a high level of occupational stress and 41% to a moderate level. Distributions were similar regarding job satisfaction: 48.5% of GPs reported being very satisfied with their job, and 10% were slightly (low) satisfied (Table 1 and Figure 2).

Occupational stress was higher among female (no significant difference for job satisfaction) and the youngest GPs (≤44yrs). Job satisfaction was higher among the youngest and the oldest GPs. They also varied by country (Figure 2, Tables 1 [Table table2]-3).

**Table 2. table2:** Association between job satisfaction and PC team composition: ordered logistic regression

		Univariate model		Multivariate model (*n* = 10 100)	
	Number	OR	95% CI	OR	95% CI
**Sex**					
Male (Ref)	7136	1		1	
Female	6004	0.89	0.83 to 0.95	**0.90**	**0.83 to 0.97**
**Age (years)**					
55–65 (Ref)	3845	1		1	
<35–44	4408	1.15	1.06 to 1.25	**1.21**	**1.09 to 1.34**
45–55	3845	1.08	0.97 to 1.16	**1.13**	**1.01 to 1.25**
>65	1728	1.56	1.40 to 1.75	**1.59**	**1.40 to 1.81**
**Area**					
Rural (Ref)	1798	1		1	
Suburb	2694	0.70	0.62 to 0.78	**0.77**	**0.67 to 0.87**
Small town	2785	0.82	0.73 to 0.93	**0.84**	**0.73 to 0.95**
City	5650	0.80	0.72 to 0.89	**0.87**	**0.77 to 0.99**
**Consultation time**					
>20 minute to≤90 minute (Ref)	2672	1		1	
≥5 minute to≤10 minute	2720	0.61	0.55 to 0.68	**0.79**	**0.68 to 0.93**
>10 minute to≤15 minute	4269	0.83	0.76 to 0.91	1.10	0.96 to 1.25
>15 minute to≤20 minute	3322	0.90	0.82 to 1.00	**1.16**	**1.01 to 1.33**
**GP workforce (FTE)**					
<0.1–1.3 (ref)	2610				
≤1.4–3	2497	1.10	0.99 to 1.23	**1.12**	**1.00 to 1.25**
≤3–5	2379	1.05	0.94 to 1.17	**1.17**	**1.04 to 1.33**
≤5–100	3075	1.07	0.96 to 1.18	**1.18**	**1.04 to 1.34**
**Practice type**					
Traditional type (Ref)	6771	1		1	
Multidisciplinary	1643	1.12	1.01 to 1.24	**1.43**	**1.26 to 1.62**
Nurse-centred	1560	0.99	0.90 to 1.11	**1.18**	**1.02 to 1.35**
Psychologist-centred	2447	1.23	1.12 to 1.34	**1.24**	**1.09 to 1.42**
Physiotherapist-centred	992	0.98	0.85 to 1.14	0.98	0.83 to 1.15
**Country**					
Australia (Ref)	500				
Canada	2569	0.49	0.40 to 0.59	**0.43**	**0.35 to 0.53**
France	1287	0.36	0.29 to 0.44	**0.36**	**0.29 to 0.45**
Germany	809	0.46	0.37 to 0.57	**0.65**	**0.51 to 0.84**
the Netherlands	788	0.75	0.60 to 0.94	0.93	0.72 to 1.19
New Zealand	503	0.71	0.56 to 0.91	**0.74**	**0.57 to 0.95**
Norway	661	0.91	0.72 to 1.16	0.84	0.65 to 1.08
Sweden^a^	2411	0.63	0.52 to 0.77	-	-
Switzerland	1095	1.43	1.15 to 1.78	**1.51**	**1.19 to 1.92**
UK	1001	0.37	0.30 to 0.46	**0.44**	**0.35 to 0.56**
US	1576	0.42	0.34 to 0.51	**0.38**	**0.31 to 0.48**

^a^Swedish GPs did not answer to the question about FTE. FTE = full-time equivalents

**Table 3.** Associations between occupational stress and PC team composition: ordered logistic regression

		Univariate model		Multivariate model (*n* = 10 088)	
	Number	OR	95% CI	OR	95% CI
**Sex**					
Male (Ref)	7136	1		1	
Female	6004	1.36	1.28 to 1.46	**1.17**	**1.08 to 1.27**
**Age (years)**					
55–65 (Ref)	3845	1		1	
35–44	4408	1.26	1.16 to 1.38	**1.12**	**1.01 to 1.25**
45–55	3845	1.23	1.12 to 1.35	**1.12**	**1.00 to 1.24**
>65	1728	0.50	0.45 to 0.56	**0.51**	**0.45 to 0.58**
**Area**					
Rural (Ref)	1798	1		1	
Suburb	2694	1.15	1.02 to 1.29	1.04	0.92 to 1.19
Small town	2785	1.19	1.06 to 1.34	1.03	0.91 to 1.17
City	5650	1.29	1.17 to 1.44	1.00	0.97 to 1.21
**Consultation time**					
>20 minute to≤90 minute (Ref)	2672	1		1	
≥5 minute to≤10 minute	2720	1.23	1.11 to 1.37	**1.76**	**1.50 to 2.06**
>10 minute to≤15 minute	4269	0.95	0.87 to 1.04	**1.31**	**1.16 to 1.48**
>15 minute to≤20 minute	3322	1.05	0.96 to 1.16	1.09	0.96 to 1.25
**GP workforce (FTE)^a,^**					
<0.1–1.3 (Ref)	2610	1			
≤1.4–3	2497	1.11	1.00 to 1.23	1.00	0.89 to 1.12
≤3–5	2379	1.20	1.07 to 1.33	0.93	0.83 to 1.05
≤5–100	3075	1.26	1.14 to 1.39	0.94	0.83 to 1.06
**Practice type**					
Traditional type (Ref)	6771	1		1	
Multidisciplinary	1643	0.85	0.77 to 0.94	**0.77**	**0.68 to 0.87**
Nurse-centred	1560	1.34	1.20 to 1.49	0.87	0.76 to 1.00
Psychologist-centred	2447	1.08	0.98 to 1.18	**0.81**	0.71 to 0.93
Physiotherapist-centred	992	0.82	0.71 to 0.95	1.04	0.88 to 1.23
**Country**					
Australia (Ref)	500	1		1	
Canada	2569	1.87	1.56 to 2.24	**2.15**	**1.77 to 2.61**
France	1287	1.42	1.17 to1.73	**1.63**	**1.32 to 2.01**
Germany	809	2.30	1.85 to 2.84	**1.78**	**1.39 to 2.28**
The Netherlands	788	1.12	0.91 to 1.39	0.86	0.68 to 1.10
New Zealand	503	1.55	1.22 to1.96	**1.49**	**1.17 to 1.91**
Norway	661	1.86	1.50 to 2.32	**2.14**	**1.69 to 2.71**
Sweden^a^	2411	4.06	1.06 to 1.58	-	-
Switzerland	1095	1.29	1.15 to 1.78	**1.45**	**1.16 to 1.81**
UK	1001	3.32	2.70 to 4.08	**2.59**	**2.06 to 3.26**
US	1576	2.41	1.99 to 2.91	**2.91**	**2.36 to 3.59**

^a^Swedish GPs did not answer the question about FTE. FTE = full-time equivalents

### Associations with PC practices’ professional composition

In our multivariate analyses, GPs’ job satisfaction was higher when they worked in multidisciplinary (OR 1.43, 95% CI = 1.26 to 1.62), psychologist-centred or nurse-centred practices (OR 1.24, 95% CI = 1.09 to 1.42 and OR 1.18, 95% CI = 1.02 to 1.35, respectively) than if they worked in traditional practicesTable 2. Stratified analyses, according to the number of FTE GPs at the practice, showed that the association between GPs’ job satisfaction and nurse-centred practices was only observed in the smallest practices (FTE <1.4) (Table 4). Job satisfaction was also higher among the youngest and oldest GPs than among those aged 55–64 years and in GPs located in rural areas (Table 2).

**Table 4.** Association between job satisfaction, occupational stress, and primary care team composition, according to GP FTEs in the practice: ordered logistic regression^a^

GP workforce (FTE)^,^	<0.1–1.4 (*n* = 2610)			≤1.4–3 (*n* = 2497)		≤3–5 (*n* = 2379)		≤5–100 (*n* = 3075)	
		**Job satisfaction**							
	**OR**		**95% CI**	**OR**	**95% CI**	**OR**	**95% CI**	**OR**	**95% CI**
**Practice type**									
Traditional type (Ref)	1		-	1	-	1	-	1	-
Multidisciplinary	1.36		0.94 to 1.99	**1.40**	**1.01 to 1.95**	**1.94**	**1.48 to 2.54**	**1.36**	**1.12 to 1.64**
Nurse-centred	**1.59**		**1.14 to 2.22**	0.91	0.68 to 1.22	1.23	0.93 to 1.61	1.16	0.89 to 1.50
Psychologist-centred	1.29		0.99 to 1.67	1.11	0.86 to 1.44	1.14	0.86 to 1.51	1.37	1.00 to 1.88
Physiotherapist-centred	0.77		0.55 to 1.08	1.24	0.89 to 1.72	0.68	0.48 to 10.97	1.19	0.85 to 1.66
		**Occupational stress**							
	**OR**		**95% CI**	**OR**	**95% CI**	**OR**	**95% CI**	**OR**	**95% CI**
**Practice type**									
Traditional type (Ref)	1		-	1	-	1	-	1	-
Multidisciplinary	**0.63**		**0.44 to 0.91**	1.00	0.72 to 1.40	**0.61**	**0.47 to 0.79**	**0.72**	**0.62 to 0.91**
Nurse-centred	0.96		0.69 to 1.32	0.95	0.71 to 1.27	0.83	0.63 to 1.09	0.79	0.61 to 1.03
Psychologist-centred	**0.75**		**0.58 to 0.97**	1.00	0.78 to 1.27	**0.76**	**0.57 to 1.00**	**0.72**	**0.53 to 0.98**
Physiotherapist-centred	0.83		0.60 to 1.15	1.27	0.92 to 1.74	1.31	0.92 to 1.87	0.89	0.64 to 1.25

^a^Models adjusted for sex, age category, area, and country. FTE: Full-time equivalents

The results were quite similar regarding occupational stress. In the multivariate analyses, GPs working in multidisciplinary (OR 0.77, 95% CI = 0.68 to 0.87) and psychologist-centred practices reported lower occupational stress (OR 0.81, 95% CI = 0.71 to 0.93) than those working in traditional practices; the results about nurse-centred practices were almost significant as well (OR 0.87, 95% CI = 0.76 to 1.00) (Table 3). These associations were observed whatever the number of FTE GPs (Table 4). In addition, occupational stress was higher among female GPs (OR 1.17, 95% CI = 1.08 to 1.27), among younger and middle-aged GPs aged 35–44 years (OR 1.12, 95% CI = 1.01 to 1.25) and GPs aged 45–54 years (OR 1.12, 95% CI = 1.00 to 1.24), and those with the shortest consultation durations between 5 minutes and 10 minutes (OR 1.76, 95% CI = 1.50 to 2.06).

## Discussion

### Summary

Our results showed that occupational stress and job satisfaction among GPs varied according to their personal characteristics, such as sex, age, and country of work, but also according to their PC practice’s organisational characteristics. Working in multidisciplinary teams was associated with higher wellbeing. This association, when compared with the traditional model, was observed in two of the models created by our typology: the multidisciplinary team model and the psychologist-centred model. It was also observed with nurse-centred practices but only for job satisfaction in the smallest practices.

### Strengths and limitations

The present study had some limitations. The first was the creation of our PC practice typology, which used a statistical construction to obtain a composite indicator. Using this kind of indicator can lead to imprecision, and the names given to the clusters were arbitrary and obviously reductive. Consequently, these results should be interpreted with the composition of the clusters in mind. In this study, we only took into account interprofessional collaboration within practices. However, interprofessional work also exists through integrated care networks and can also be a source of wellbeing. The self-reported nature of the outcomes make them subject to reporting biases. In addition, they were assessed using a single question rather than a validated questionnaire. This might have led to an overestimation of their frequency. Furthermore, there was a socio-cultural component inherent in these data (perception of stress and dissatisfaction may be differ according to the country). Survey participation rates varied from 19%–47%. This could have led to an overestimation of frequency of job stress and dissatisfaction if the main focus of the survey was wellbeing at work. Thus, we believe that this bias was limited. However, data were weighted to account for differential non-response rates, according to known demographic parameters, thus limiting the potential for selection bias as well. Finally, this was a cross-sectional study and, therefore, the observed statistical associations are not necessarily causal.

The study had several strengths, notably in terms of sample size and international comparisons, thanks to standardised methodology and questionnaire. The circular phenomenon of data (when independent and dependent variables are self-perceived variables) was limited because the typology was built on factual variables. To the best of our knowledge, it is the largest study performed in the domain of stress and dissatisfaction at work among GPs.

### Comparison with existing literature

These results confirmed previous studies in the literature showing that, despite some challenges linked to interprofessional collaboration, the inclusion of professionals other than GPs in PC practices might be a source of overall wellbeing.^36–38^ The two organisational models in our study that were systematically associated with better wellbeing shared the characteristic of including several kinds of healthcare professionals. The psychologist-centred model included not just psychologists but also different kinds of nurses, nutritionists, and physiotherapists. Furthermore, these two organisational models were also those that most often included psychologists. We can hypothesise that working with psychologists in PC practices provides GPs with a degree of ease. Indeed, patients suffering from mental health disorders often generate a high management burden for GPs (that is, frequent and longer consultations, social problems to address).^39^ Being able to easily refer these patients to a psychologist in the same location probably lightens this burden and, therefore, lowers the GP’s occupational stress.^40–42^ This interpretation is in line with theoretical models of stress not only on the role of workload but also on that of social support.^16,17^ Another potential interpretation is that practices incorporating psychologists are more psychosocially oriented and, therefore, more in line with patients’ needs, given the high prevalence of mental health symptoms in the population. This may also be a source of physicians’ wellbeing referring to the meaning of work, which is often very important to the doctor.^10^ The nurse-centred practice model was associated with higher reported satisfaction among GPs, but only in very small practices (including mainly solo practices). Integrating a nurse into a traditional practice is often the first step towards team-based care. It seems that this step alone is sufficient to improve GPs’ satisfaction. However, for the biggest practices, it seemed that this was not the best organisational model to adopt and that other combinations of PC professionals were more relevant in terms of occupational wellbeing.^22,24^

Other studies among PC professionals have already shown lower wellbeing among women.^7,43–45^ The main reason given was the difficulty of balancing private and professional life,^44,45^ but the link with gender may vary depending on the reason for satisfaction.^43^ With regard to age-related results, in a previous study based on the 2015 Commonwealth Fund International Health Policy Survey of Primary Care Physicians, we have already found the same type of associations, namely that lower satisfaction is observed among middle-aged doctors. In fact, the last age class (where satisfaction is higher) is >65 years, and this is probably owing to a selection phenomenon. Those who stay at work are the happiest.^30^

### Implications for research and practice

These results are interesting and relevant for public health authorities and healthcare policies. The development of PC teams is one of the most frequently adopted strategies by the many Western countries attempting to strengthen PC. It is a strategy that has shown positive results in terms of the access, coordination, continuity, and comprehensiveness of PC (the four pillars of PC according to Starfield).^46^ Positive effects on patients’ clinical outcomes have also been demonstrated.^12,13^ In the current context of shortages of GPs and difficulties recruiting new physicians to the profession, such positive results could have a considerable impact on younger generations of physicians, too, which will be critical for the sustainability of health systems.^2^ Additionally, these results could be used to highlight the benefits of team-based PC practice models and advocate for their adoption.

The next step in analysing the links between PC teams and professionals’ wellbeing could be to determine the optimal distribution of roles among the different kinds of professionals, to deliver the most appropriate care and to best satisfy healthcare staff. The present study did not allow for such an analysis as the numbers of each type of professional were not collected. A more in-depth study should look at the efficiency of care processes, patients’ clinical outcomes, and professionals’ wellbeing. With this in mind, it’s worth noting that this study focuses on the presence (and the type) of multiprofessional teams. They do not allow us to explore whether there is real interprofessional collaboration and how it works (task transfer, task delegation, and so on), which is a critical issue in this field. Finally, if the wellbeing of physicians is fundamental, so too is that of other professionals. Several studies have shown high levels of satisfaction among nurses working in PC teams. Studies are much rarer for other professionals compared with GPs.

The preservation of healthcare professionals’ wellbeing is a critical issue for the sustainability of PC systems and should not be neglected. These results suggest optimistic perspectives.

## Data

The dataset relied on in this article is available from the corresponding author on reasonable request.
